# Supplementary material for: Geographical Distribution and Genetic Diversity of Bank Vole Hepaciviruses in Europe
Source: Viruses. 2021 Jun 28;13(7):1258. doi: 10.3390/v13071258 (PMC8310187; doi:10.3390/v13071258)
Supplement: Supplementary file 1 [file viruses-13-01258-s001.zip › viruses-1244550-supplementary.pdf]

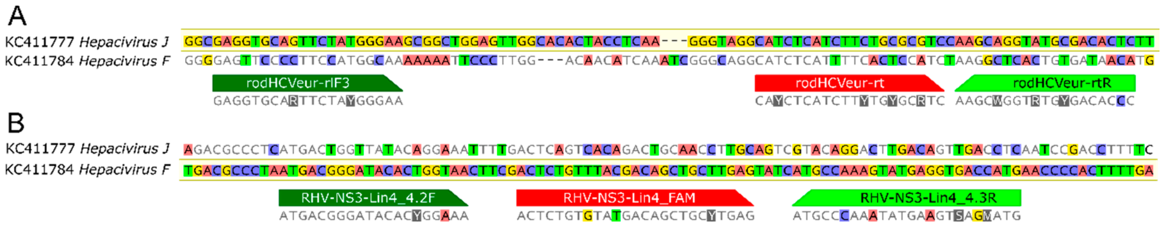

Figure S1: Primer and probe sequences of the two RT-qPCR assays rodHCVeur (A) and RHV-NS3-Line4 (B) used for BvHV detection compared to corresponding homologous and heterologous prototype strains of the *Hepacivirus J* and *Hepacivirus F* species. Highlighted are mismatches and ambiguities in comparison to the reference sequence (yellow). Abbreviations: **M**, A or C; **R**, A or G; **S**, G or C; **W**, A or T; **Y**, T or C.

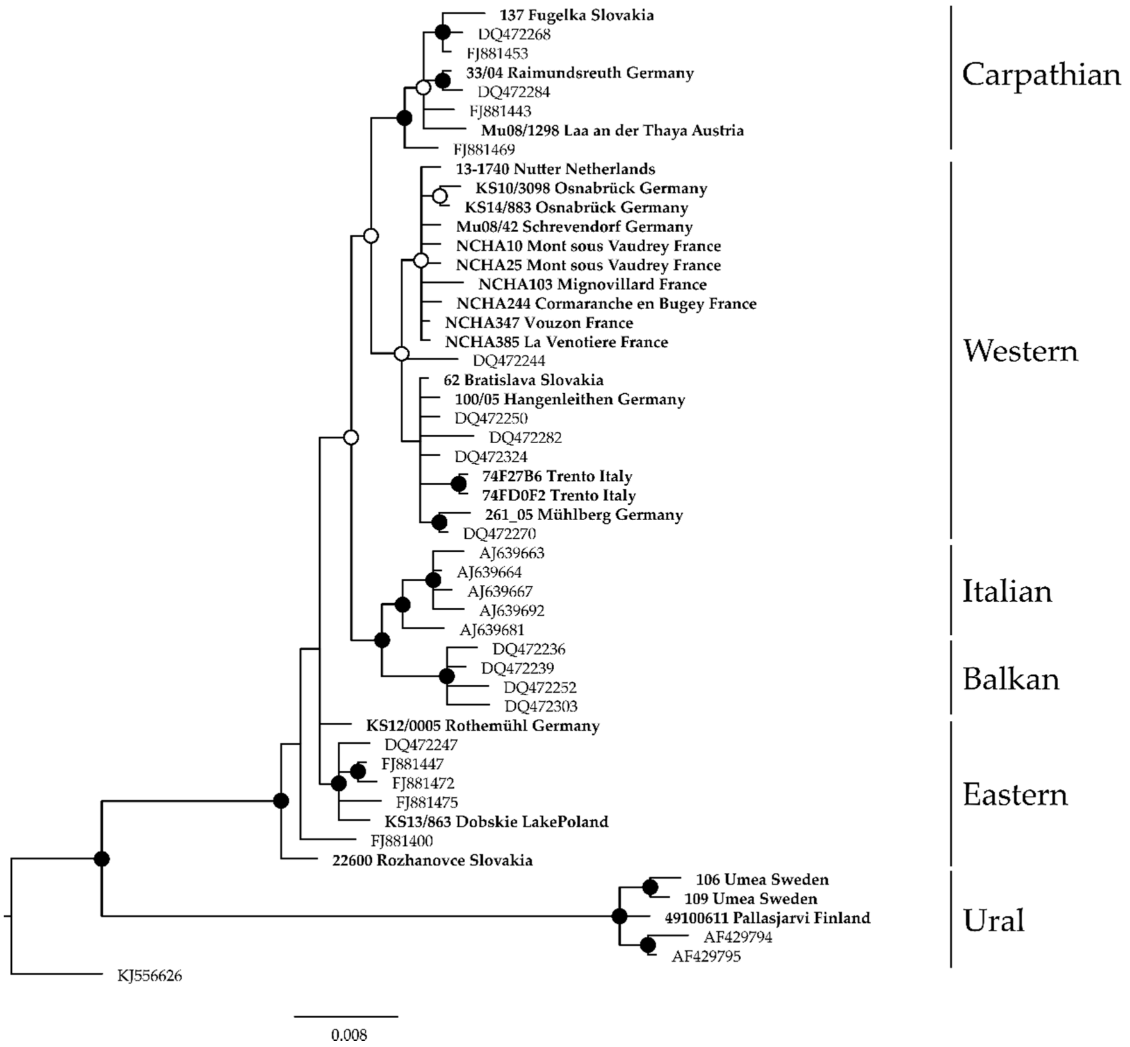

Figure S2: Phylogenetic relationships of partial cyt *b* gene sequences (764 nt). Sequences generated during this study are highlighted in bold. Circle colour at nodes indicates posterior Bayesian probabilities: black: probability exceeds 90 %; white: probability exceeds 70 %. Reference sequences obtained from GenBank are indicated by their accession numbers.

Table S1: Sequences of primers and probes used in this study.

Primer	Sequence 5′-3′	Source
**BvHV-Screening**		
rodHCVeur-rlF3	GAG GTG CAR TTC TAY GGGAA	[1]
rodHCVeur-rtR	GGG TGT CRC AYA CCW GCT T	[1]
rodHCVeur-rt	VIC-CAY CTC ATC TTY TGY GCR TC-MGBNFQ	[1]
RHV-NS3-Line4_4.2-F	ATG ACG GGA TAC ACY GGA AA	[2]
RHV-NS3-Line4_4.3-R	CAT KGT SAC TTC ATA TTT GGG CAT	[2]
RHV-NS3-Line4_4-FAM	FAM-ACT CTG TGT ATG ACA GCT GCY TGA G	[2]
**EGFP-Mix1 – internal Control**		
EGFP-1-F	GAC CAC TAC CAG CAG AAC AC	[3]
EGFP-2-R	GAA CTC CAG CAG GAC CAT G	[3]
EGFP-Probe1	HEX-AGC ACC CAGTCC GCC CTG AGC A-BHQ1	[3]
**BvHV-NS3**		
rodHV_NS3_932F	CGG AAA GAC CAC YAA RGT GCC	this study
rodHV_NS3_1567R	AAG TTT CCD GTG TAD CCV GTC AT	this study

Abbreviations: **D**, A or G or T; **K**, G or T; **M**, A or C; **R**, A or G; **S**, G or C; **V**, A or C or G; **W**, A or T; **Y**, T or C.

Table S2: Semiquantitative genome loads of all BvHV RNA positive animals. *Hepacivirus J* and *Hepacivirus F* were detected by RT-qPCR assay rodHCVeur and RHV-NS3-Line4, respectively.

Country	Sample ID	Trapping site	Species	*Hepacivirus J*	*Hepacivirus F*	Bank vole evolutionary lineage
Germany	KS10/3254	Ahlhorn	*Myodes glareolus*	+++	neg	nd
Germany	KS10/2185	Billerbeck	*Myodes glareolus*	++++	neg	nd
Germany	KS10/2176	Billerbeck	*Myodes glareolus*	+++	neg	nd
Germany	KS10/3034	Billerbeck	*Myodes glareolus*	+++	neg	nd
Germany	KS10/2094	Billerbeck	*Myodes glareolus*	+++	neg	nd
Germany	KS10/2197	Billerbeck	*Myodes glareolus*	+++	neg	nd
Germany	KS10/2234	Billerbeck	*Myodes glareolus*	+++	neg	nd
Germany	KS10/2709	Billerbeck	*Myodes glareolus*	+++	neg	nd
Germany	KS10/2688	Billerbeck	*Myodes glareolus*	+++	neg	nd
Germany	KS10/2202	Billerbeck	*Myodes glareolus*	+++	neg	nd
Germany	KS10/3022	Billerbeck	*Myodes glareolus*	+++	neg	nd
Germany	KS10/2160	Billerbeck	*Myodes glareolus*	+++	neg	nd
Germany	KS10/2236	Billerbeck	*Myodes glareolus*	+++	neg	nd
Germany	KS10/2214	Billerbeck	*Myodes glareolus*	+++	neg	nd
Germany	KS10/2172	Billerbeck	*Myodes glareolus*	+++	neg	nd
Germany	KS10/2045	Billerbeck	*Myodes glareolus*	+++	neg	nd
Germany	KS10/2099	Billerbeck	*Myodes glareolus*	+++	neg	nd
Germany	KS10/1976	Billerbeck	*Myodes glareolus*	+++	neg	nd
Germany	KS10/2015	Billerbeck	*Myodes glareolus*	+++	neg	nd
Germany	KS11/2240	Billerbeck	*Myodes glareolus*	+++	neg	nd
Germany	KS11/1250	Billerbeck	*Myodes glareolus*	+++	neg	nd
Germany	KS10/2677	Billerbeck	*Myodes glareolus*	+++	neg	nd
Germany	KS10/2609	Billerbeck	*Myodes glareolus*	+++	neg	nd
Germany	KS10/2035	Billerbeck	*Myodes glareolus*	+++	neg	nd
Germany	KS11/2251	Billerbeck	*Myodes glareolus*	+++	neg	nd
Germany	KS10/2195	Billerbeck	*Myodes glareolus*	+++	neg	nd
Germany	KS10/2002	Billerbeck	*Myodes glareolus*	+++	neg	nd
Germany	KS11/1922	Billerbeck	*Myodes glareolus*	+++	neg	nd
Germany	KS10/2660	Billerbeck	*Myodes glareolus*	+++	neg	nd
Germany	KS10/2701	Billerbeck	*Myodes glareolus*	+++	neg	nd
Germany	KS10/2027	Billerbeck	*Myodes glareolus*	+++	neg	nd
Germany	KS10/2186	Billerbeck	*Myodes glareolus*	+++	neg	nd
Germany	KS11/2233	Billerbeck	*Myodes glareolus*	+++	neg	nd
Germany	KS10/2710	Billerbeck	*Myodes glareolus*	++	neg	nd
Germany	KS10/1973	Billerbeck	*Myodes glareolus*	++	neg	nd
Germany	KS10/2239	Billerbeck	*Myodes glareolus*	++	neg	nd
Germany	KS10/2000	Billerbeck	*Myodes glareolus*	++	neg	nd
Germany	KS10/2225	Billerbeck	*Myodes glareolus*	++	neg	nd
Germany	KS10/2625	Billerbeck	*Myodes glareolus*	++	neg	nd
Germany	KS10/2012	Billerbeck	*Myodes glareolus*	++	neg	nd
Germany	KS10/2184	Billerbeck	*Myodes glareolus*	++	neg	nd
Germany	KS10/2685	Billerbeck	*Myodes glareolus*	++	neg	nd
Germany	KS10/2175	Billerbeck	*Myodes glareolus*	++	neg	nd
Germany	KS10/2689	Billerbeck	*Myodes glareolus*	++	neg	nd
Germany	KS11/1925	Billerbeck	*Myodes glareolus*	++	neg	nd
Germany	KS10/2657	Billerbeck	*Myodes glareolus*	++	neg	nd
Germany	KS10/1977	Billerbeck	*Myodes glareolus*	++	neg	nd
Germany	KS10/2673	Billerbeck	*Myodes glareolus*	++	neg	nd
Germany	KS10/1970	Billerbeck	*Myodes glareolus*	++	neg	nd
Germany	KS10/2009	Billerbeck	*Myodes glareolus*	++	neg	nd
Germany	KS10/2026	Billerbeck	*Myodes glareolus*	++	neg	nd
Germany	KS10/2010	Billerbeck	*Myodes glareolus*	++	neg	nd
Germany	KS10/2245	Billerbeck	*Myodes glareolus*	++	neg	nd
Germany	KS10/2696	Billerbeck	*Myodes glareolus*	++	neg	nd
Germany	KS10/2714	Billerbeck	*Myodes glareolus*	++	neg	nd
Germany	KS10/2023	Billerbeck	*Myodes glareolus*	++	neg	nd
Germany	KS10/1994	Billerbeck	*Myodes glareolus*	++	neg	nd
Germany	KS10/2693	Billerbeck	*Myodes glareolus*	++	neg	nd
Germany	KS10/2240	Billerbeck	*Myodes glareolus*	+	neg	nd
Germany	KS10/2033	Billerbeck	*Myodes glareolus*	+	neg	nd
Germany	KS10/2705	Billerbeck	*Myodes glareolus*	+	neg	nd
Germany	KS10/2237	Billerbeck	*Myodes glareolus*	+	neg	nd
Germany	KS11/1910	Billerbeck	*Myodes glareolus*	+	neg	nd
Germany	KS10/2204	Billerbeck	*Myodes glareolus*	+	neg	nd
Germany	KS10/1999	Billerbeck	*Myodes glareolus*	+	neg	nd
Germany	KS12/1778	Billerbeck	*Myodes glareolus*	+++	neg	nd

Germany	KS12/1821	Billerbeck	*Myodes glareolus*	++	neg	nd
Germany	KS12/1794	Billerbeck	*Myodes glareolus*	+++	neg	nd
Germany	KS10/2219	Billerbeck	*Myodes glareolus*	neg	++++	nd
Germany	KS10/2173	Billerbeck	*Myodes glareolus*	neg	++++	nd
Germany	KS10/2180	Billerbeck	*Myodes glareolus*	neg	++++	nd
Germany	KS10/2104	Billerbeck	*Myodes glareolus*	neg	+++	nd
Germany	KS10/2167	Billerbeck	*Myodes glareolus*	neg	+++	nd
Germany	KS10/2179	Billerbeck	*Myodes glareolus*	neg	+++	nd
Germany	KS10/2101	Billerbeck	*Myodes glareolus*	neg	++	nd
Germany	KS10/2100	Billerbeck	*Myodes glareolus*	neg	++	nd
Germany	BH6	Bremerhaven	*Myodes glareolus*	++++	neg	nd
Germany	BH17	Bremerhaven	*Myodes glareolus*	+++	neg	nd
Germany	BH9	Bremerhaven	*Myodes glareolus*	+++	neg	nd
Germany	41/04	Falkenstein	*Myodes glareolus*	neg	++++	nd
Germany	36/04	Falkenstein	*Myodes glareolus*	neg	++++	nd
Germany	KS11/1113	Gotha	*Myodes glareolus*	++++	++++	nd
Germany	KS10/1602	Gotha	*Myodes glareolus*	++++	neg	nd
Germany	KS11/2126	Gotha	*Myodes glareolus*	++++	++++	nd
Germany	KS11/1894	Gotha	*Myodes glareolus*	++++	++++	nd
Germany	KS11/0274	Gotha	*Myodes glareolus*	++++	++++	nd
Germany	KS12/1397	Gotha	*Myodes glareolus*	++++	++++	nd
Germany	KS11/2105	Gotha	*Myodes glareolus*	+++	++++	nd
Germany	KS11/1880	Gotha	*Myodes glareolus*	+++	++++	nd
Germany	KS12/1386	Gotha	*Myodes glareolus*	+++	++++	nd
Germany	KS11/2100	Gotha	*Myodes glareolus*	+++	++++	nd
Germany	KS11/0221	Gotha	*Myodes glareolus*	+++	++++	nd
Germany	KS11/2088	Gotha	*Myodes glareolus*	+++	++++	nd
Germany	KS11/1891	Gotha	*Myodes glareolus*	+++	++++	nd
Germany	KS11/0235	Gotha	*Myodes glareolus*	+++	+++	nd
Germany	KS11/2089	Gotha	*Myodes glareolus*	+++	++++	nd
Germany	KS11/0211	Gotha	*Myodes glareolus*	+++	neg	nd
Germany	KS11/2134	Gotha	*Myodes glareolus*	+++	++++	nd
Germany	KS10/1486	Gotha	*Myodes glareolus*	+++	+++	nd
Germany	KS11/1096	Gotha	*Myodes glareolus*	+++	++++	nd
Germany	KS10/1580	Gotha	*Myodes glareolus*	+++	++++	nd
Germany	KS11/2133	Gotha	*Myodes glareolus*	+++	++++	nd
Germany	KS11/0203	Gotha	*Myodes glareolus*	+++	+++	nd
Germany	KS11/1897	Gotha	*Myodes glareolus*	+++	++++	nd
Germany	KS11/2097	Gotha	*Myodes glareolus*	+++	++++	nd
Germany	KS10/2789	Gotha	*Myodes glareolus*	+++	+++	nd
Germany	KS10/1481	Gotha	*Myodes glareolus*	+++	++++	nd
Germany	KS11/1881	Gotha	*Myodes glareolus*	+++	++++	nd
Germany	KS11/0276	Gotha	*Myodes glareolus*	+++	+++	nd
Germany	KS11/1104	Gotha	*Myodes glareolus*	+++	++++	nd
Germany	KS10/1576	Gotha	*Myodes glareolus*	+++	+++	nd
Germany	KS10/1482	Gotha	*Myodes glareolus*	+++	++++	nd
Germany	KS10/2717	Gotha	*Myodes glareolus*	+++	++++	nd
Germany	KS11/0223	Gotha	*Myodes glareolus*	+++	+++	nd
Germany	KS11/1892	Gotha	*Myodes glareolus*	+++	+++	nd
Germany	KS11/2127	Gotha	*Myodes glareolus*	+++	++++	nd
Germany	KS11/1889	Gotha	*Myodes glareolus*	+++	++++	nd
Germany	KS12/1388	Gotha	*Myodes glareolus*	+++	++++	nd
Germany	KS10/2763	Gotha	*Myodes glareolus*	+++	++++	nd
Germany	KS10/2968	Gotha	*Myodes glareolus*	+++	neg	nd
Germany	KS11/0121	Gotha	*Myodes glareolus*	++	+++	nd
Germany	KS10/1575	Gotha	*Myodes glareolus*	++	+++	nd
Germany	KS11/1902	Gotha	*Myodes glareolus*	++	++++	nd
Germany	KS11/1869	Gotha	*Myodes glareolus*	++	++++	nd
Germany	KS12/1401	Gotha	*Myodes glareolus*	++	++++	nd
Germany	KS11/1903	Gotha	*Myodes glareolus*	++	+++	nd
Germany	KS11/0281	Gotha	*Myodes glareolus*	++	++	nd
Germany	KS10/2736	Gotha	*Myodes glareolus*	++	++	nd
Germany	KS11/0253	Gotha	*Myodes glareolus*	++	++	nd
Germany	KS10/2722	Gotha	*Myodes glareolus*	+	neg	nd
Germany	KS12/1405	Gotha	*Myodes glareolus*	+	++	nd
Germany	KS12/1396	Gotha	*Myodes glareolus*	+	++	nd
Germany	KS12/1399	Gotha	*Myodes glareolus*	+	+	nd
Germany	KS10/1579	Gotha	*Myodes glareolus*	+	neg	nd
Germany	KS12/1385	Gotha	*Myodes glareolus*	++++	++++	nd
Germany	KS12/1379	Gotha	*Myodes glareolus*	+++	++++	nd
Germany	KS12/1398	Gotha	*Myodes glareolus*	+++	++++	nd
Germany	KS10/2958	Gotha	*Myodes glareolus*	neg	++++	nd
Germany	KS11/2149	Gotha	*Myodes glareolus*	neg	++++	nd

Germany	KS10/2972	Gotha	*Myodes glareolus*	neg	+++	nd
Germany	KS10/2978	Gotha	*Myodes glareolus*	neg	+++	nd
Germany	KS10/1587	Gotha	*Myodes glareolus*	neg	+++	nd
Germany	KS10/1595	Gotha	*Myodes glareolus*	neg	+++	nd
Germany	KS10/2806	Gotha	*Myodes glareolus*	neg	+++	nd
Germany	KS10/1604	Gotha	*Myodes glareolus*	neg	++	nd
Germany	KS12/1393	Gotha	*Myodes glareolus*	neg	++	nd
Germany	KS10/1581	Gotha	*Myodes glareolus*	neg	+	nd
Germany	20/04	Hangenleithen	*Myodes glareolus*	neg	++++	nd
Germany	100/05	Hangenleithen	*Myodes glareolus*	neg	++++	Western
Germany	14/04	Hangenleithen	*Myodes glareolus*	neg	++++	nd
Germany	KS12/0192	Jasnitz	*Myodes glareolus*	+++	neg	nd
Germany	KS12/0195	Jasnitz	*Myodes glareolus*	++	neg	nd
Germany	KS12/0206	Jasnitz	*Myodes glareolus*	++	neg	nd
Germany	KS12/0247	Jasnitz	*Myodes glareolus*	+	neg	nd
Germany	KS12/0196	Jasnitz	*Myodes glareolus*	+	neg	nd
Germany	KS10/2446	Jeeser	*Myodes glareolus*	++++	neg	nd
Germany	KS11/2175	Jeeser	*Myodes glareolus*	+++	neg	nd
Germany	KS12/1426	Jeeser	*Myodes glareolus*	+++	+	nd
Germany	KS11/2183	Jeeser	*Myodes glareolus*	+++	neg	nd
Germany	KS10/3652	Jeeser	*Myodes glareolus*	+++	neg	nd
Germany	KS11/2158	Jeeser	*Myodes glareolus*	+++	neg	nd
Germany	KS11/2157	Jeeser	*Myodes glareolus*	+++	neg	nd
Germany	KS11/2170	Jeeser	*Myodes glareolus*	+++	neg	nd
Germany	KS11/0004	Jeeser	*Myodes glareolus*	+++	neg	nd
Germany	KS10/3632	Jeeser	*Myodes glareolus*	+++	neg	nd
Germany	KS10/3623	Jeeser	*Myodes glareolus*	+++	neg	nd
Germany	KS10/3628	Jeeser	*Myodes glareolus*	+++	neg	nd
Germany	KS10/1445	Jeeser	*Myodes glareolus*	+++	neg	nd
Germany	KS11/0005	Jeeser	*Myodes glareolus*	++	neg	nd
Germany	KS10/3618	Jeeser	*Myodes glareolus*	++	neg	nd
Germany	KS10/3601	Jeeser	*Myodes glareolus*	++	neg	nd
Germany	KS11/2192	Jeeser	*Myodes glareolus*	++	neg	nd
Germany	KS10/1455	Jeeser	*Myodes glareolus*	++	neg	nd
Germany	KS10/2368	Jeeser	*Myodes glareolus*	++	neg	nd
Germany	KS10/1448	Jeeser	*Myodes glareolus*	++	++++	nd
Germany	KS11/0015	Jeeser	*Myodes glareolus*	++	neg	nd
Germany	KS10/3611	Jeeser	*Myodes glareolus*	++	neg	nd
Germany	KS10/2325	Jeeser	*Myodes glareolus*	++	neg	nd
Germany	KS11/2159	Jeeser	*Myodes glareolus*	++	neg	nd
Germany	KS11/2196	Jeeser	*Myodes glareolus*	++	neg	nd
Germany	KS10/1454	Jeeser	*Myodes glareolus*	++	neg	nd
Germany	KS10/2401	Jeeser	*Myodes glareolus*	++	neg	nd
Germany	KS10/1452	Jeeser	*Myodes glareolus*	++	neg	nd
Germany	KS11/2172	Jeeser	*Myodes glareolus*	++	neg	nd
Germany	KS10/2334	Jeeser	*Myodes glareolus*	++	neg	nd
Germany	KS10/2403	Jeeser	*Myodes glareolus*	+	neg	nd
Germany	KS10/2414	Jeeser	*Myodes glareolus*	+	neg	nd
Germany	KS10/2408	Jeeser	*Myodes glareolus*	+	neg	nd
Germany	KS10/3604	Jeeser	*Myodes glareolus*	+	neg	nd
Germany	KS10/3588	Jeeser	*Myodes glareolus*	+	neg	nd
Germany	KS10/3610	Jeeser	*Myodes glareolus*	+	neg	nd
Germany	KS12/1435	Jeeser	*Myodes glareolus*	++++	neg	nd
Germany	KS12/1432	Jeeser	*Myodes glareolus*	+++	neg	nd
Germany	KS12/1422	Jeeser	*Myodes glareolus*	+++	neg	nd
Germany	KS12/1412	Jeeser	*Myodes glareolus*	++	neg	nd
Germany	KS12/1436	Jeeser	*Myodes glareolus*	++	neg	nd
Germany	KS14/0026	Jeeser	*Myodes glareolus*	neg	++++	nd
Germany	KS11/2631	Lucka (bei Groitzsch)	*Myodes glareolus*	+++	neg	nd
Germany	KS11/2632	Lucka (bei Groitzsch)	*Myodes glareolus*	++	neg	nd
Germany	KS11/2613	Lucka (bei Groitzsch)	*Myodes glareolus*	+	neg	nd
Germany	KS11/2573	Lucka (bei Groitzsch)	*Myodes glareolus*	neg	+++	nd
Germany	261/05	Mühlberg, Spiegelau	*Myodes glareolus*	neg	++++	Western
Germany	10/04	Mutzenwinkel	*Myodes glareolus*	neg	++++	nd
Germany	KS10/3049	Oberndorf (bei Hemmoor)	*Myodes glareolus*	neg	+++	nd
Germany	KS10/3039	Oberndorf (bei Hemmoor)	*Myodes glareolus*	neg	+++	nd
Germany	KS14/833	Osnabrück	*Myodes glareolus*	++++	++++	Western
Germany	KS11/2665	Osnabrück	*Myodes glareolus*	++++	+++	nd
Germany	KS11/2670	Osnabrück	*Myodes glareolus*	++++	++	nd
Germany	KS11/2668	Osnabrück	*Myodes glareolus*	++++	neg	nd
Germany	KS11/2644	Osnabrück	*Myodes glareolus*	++++	neg	nd
Germany	KS10/3089	Osnabrück	*Myodes glareolus*	++++	neg	nd
Germany	KS11/2669	Osnabrück	*Myodes glareolus*	+++	neg	nd

Germany	KS10/3117	Osnabrück	*Myodes glareolus*	+++	neg	nd
Germany	KS11/2680	Osnabrück	*Myodes glareolus*	+++	neg	nd
Germany	KS10/3098	Osnabrück	*Myodes glareolus*	+++	++++	Western
Germany	KS10/3119	Osnabrück	*Myodes glareolus*	++	neg	nd
Germany	KS10/3160	Osnabrück	*Myodes glareolus*	neg	++	nd
Germany	KS14/766	Osnabrück	*Myodes glareolus*	neg	++++	nd
Germany	KS14/778	Osnabrück	*Myodes glareolus*	neg	++++	nd
Germany	KS14/724	Osnabrück	*Myodes glareolus*	neg	++++	nd
Germany	33/04	Raimundsreuth	*Myodes glareolus*	neg	++++	Carpathian
Germany	26/04	Raimundsreuth	*Myodes glareolus*	neg	++++	nd
Germany	29/04	Raimundsreuth	*Myodes glareolus*	neg	(+)	nd
Germany	KS12/05	Rothemühl	*Myodes glareolus*	neg	++++	Eastern
Germany	KS12/02	Rothemühl	*Myodes glareolus*	neg	++++	nd
Germany	Mu/08/0042	Schrevendorf	*Myodes glareolus*	++++	neg	Western
Germany	Mu/08/0036	Schrevendorf	*Myodes glareolus*	++++	neg	nd
Germany	Mu/08/0058	Schrevendorf	*Myodes glareolus*	+++	neg	nd
Germany	Mu/08/0040	Schrevendorf	*Myodes glareolus*	+++	neg	nd
Germany	Mu/08/0041	Schrevendorf	*Myodes glareolus*	+++	neg	nd
Germany	Mu/08/0051	Schrevendorf	*Myodes glareolus*	+++	neg	nd
Germany	Mu/08/0054	Schrevendorf	*Myodes glareolus*	++++	neg	nd
Germany	Mu/08/0053	Schrevendorf	*Myodes glareolus*	+++	neg	nd
Germany	Mu/08/0044	Schrevendorf	*Myodes glareolus*	+++	neg	nd
Germany	Mu/08/0045	Schrevendorf	*Myodes glareolus*	neg	+++	nd
Germany	Mu/08/0037	Schrevendorf	*Myodes glareolus*	neg	++	nd
Germany	TM148	Tussenhausen	*Myodes glareolus*	neg	++++	nd
Germany	TM154	Tussenhausen	*Myodes glareolus*	neg	++++	nd
Germany	TM151	Tussenhausen	*Myodes glareolus*	neg	++++	nd
Germany	TM149	Tussenhausen	*Myodes glareolus*	neg	+	nd
Germany	KS11/1962	Weissach	*Myodes glareolus*	+++	neg	nd
Germany	KS10/1056	Weissach	*Myodes glareolus*	+++	neg	nd
Germany	KS11/1973	Weissach	*Myodes glareolus*	+++	neg	nd
Germany	KS11/2026	Weissach	*Myodes glareolus*	+++	neg	nd
Germany	KS10/1098	Weissach	*Myodes glareolus*	+++	neg	nd
Germany	KS10/1906	Weissach	*Myodes glareolus*	++	neg	nd
Germany	KS11/1971	Weissach	*Myodes glareolus*	++	neg	nd
Germany	KS10/3478	Weissach	*Myodes glareolus*	++	neg	nd
Germany	KS11/1981	Weissach	*Myodes glareolus*	++	neg	nd
Germany	KS10/3455	Weissach	*Myodes glareolus*	++	neg	nd
Germany	KS10/1871	Weissach	*Myodes glareolus*	++	neg	nd
Germany	KS10/1128	Weissach	*Myodes glareolus*	++	neg	nd
Germany	KS12/1688	Weissach	*Myodes glareolus*	++	neg	nd
Germany	KS10/1126	Weissach	*Myodes glareolus*	++	neg	nd
Germany	KS10/1142	Weissach	*Myodes glareolus*	++	neg	nd
Germany	KS10/1099	Weissach	*Myodes glareolus*	++	neg	nd
Germany	KS12/1702	Weissach	*Myodes glareolus*	++	neg	nd
Germany	KS10/1025	Weissach	*Myodes glareolus*	++	neg	nd
Germany	KS12/1696	Weissach	*Myodes glareolus*	++	neg	nd
Germany	KS10/0986	Weissach	*Myodes glareolus*	++	neg	nd
Germany	KS10/1855	Weissach	*Myodes glareolus*	++	neg	nd
Germany	KS10/1846	Weissach	*Myodes glareolus*	++	neg	nd
Germany	KS10/1926	Weissach	*Myodes glareolus*	++	neg	nd
Germany	KS10/1007	Weissach	*Myodes glareolus*	++	neg	nd
Germany	KS10/3472	Weissach	*Myodes glareolus*	++	neg	nd
Germany	KS10/3558	Weissach	*Myodes glareolus*	++	neg	nd
Germany	KS10/1089	Weissach	*Myodes glareolus*	++	neg	nd
Germany	KS10/1065	Weissach	*Myodes glareolus*	++	neg	nd
Germany	KS10/1109	Weissach	*Myodes glareolus*	++	neg	nd
Germany	KS10/1038	Weissach	*Myodes glareolus*	++	neg	nd
Germany	KS10/1055	Weissach	*Myodes glareolus*	++	neg	nd
Germany	KS10/0981	Weissach	*Myodes glareolus*	++	neg	nd
Germany	KS12/1740	Weissach	*Myodes glareolus*	++	neg	nd
Germany	KS10/0997	Weissach	*Myodes glareolus*	++	neg	nd
Germany	KS10/1093	Weissach	*Myodes glareolus*	++	neg	nd
Germany	KS12/1768	Weissach	*Myodes glareolus*	+++	neg	nd
Germany	KS12/1724	Weissach	*Myodes glareolus*	+++	+++	nd
Germany	KS12/1684	Weissach	*Myodes glareolus*	+++	neg	nd
Germany	KS12/1719	Weissach	*Myodes glareolus*	+++	neg	nd
Germany	KS12/1682	Weissach	*Myodes glareolus*	++	neg	nd
Germany	KS12/1769	Weissach	*Myodes glareolus*	++	neg	nd
Germany	KS10/1035	Weissach	*Myodes glareolus*	neg	++++	nd
Germany	KS12/1698	Weissach	*Myodes glareolus*	neg	++	nd
Germany	KS12/1700	Weissach	*Myodes glareolus*	neg	+	nd
Germany	KS11/1428	Wolbrechtshausen	*Myodes glareolus*	+++	neg	nd

Germany	258/05	Wolfertschlag, Eppenschlag	*Myodes glareolus*	neg	++++	nd
Finland	49100611	Pallasjarvi	*Myodes rutilus*	neg	+++	Ural
France	NCHA000246	Cormaranche-en-Bugey	*Myodes glareolus*	++++	++	nd
France	NCHA000271	Cormaranche-en-Bugey	*Myodes glareolus*	++++	neg	nd
France	NCHA000250	Cormaranche-en-Bugey	*Myodes glareolus*	++++	neg	nd
France	NCHA000272	Cormaranche-en-Bugey	*Myodes glareolus*	++++	neg	nd
France	NCHA000241	Cormaranche-en-Bugey	*Myodes glareolus*	++++	neg	nd
France	NCHA000263	Cormaranche-en-Bugey	*Myodes glareolus*	++++	neg	nd
France	NCHA000278	Cormaranche-en-Bugey	*Myodes glareolus*	++++	++	nd
France	NCHA000269	Cormaranche-en-Bugey	*Myodes glareolus*	++++	neg	nd
France	NCHA000264	Cormaranche-en-Bugey	*Myodes glareolus*	++++	neg	nd
France	NCHA000244	Cormaranche-en-Bugey	*Myodes glareolus*	+++	++	Western
France	NCHA000252	Cormaranche-en-Bugey	*Myodes glareolus*	+++	neg	nd
France	NCHA000262	Cormaranche-en-Bugey	*Myodes glareolus*	+++	neg	nd
France	NCHA000235	Cormaranche-en-Bugey	*Myodes glareolus*	neg	+++	nd
France	NCHA000275	Cormaranche-en-Bugey	*Myodes glareolus*	neg	++	nd
France	NCHA000412	La Venotiere	*Myodes glareolus*	++++	neg	nd
France	NCHA000410	La Venotiere	*Myodes glareolus*	++++	+++	nd
France	NCHA000401	La Venotiere	*Myodes glareolus*	++++	neg	nd
France	NCHA000389	La Venotiere	*Myodes glareolus*	++++	+++	nd
France	NCHA000382	La Venotiere	*Myodes glareolus*	++++	neg	nd
France	NCHA000385	La Venotiere	*Myodes glareolus*	++++	neg	Western
France	NCHA000383	La Venotiere	*Myodes glareolus*	++++	neg	nd
France	NCHA000393	La Venotiere	*Myodes glareolus*	++++	neg	nd
France	NCHA000409	La Venotiere	*Myodes glareolus*	++	++++	nd
France	NCHA000403	La Venotiere	*Myodes glareolus*	++	+++	nd
France	NCHA000416	La Venotiere	*Myodes glareolus*	neg	++++	nd
France	NCHA000396	La Venotiere	*Myodes glareolus*	neg	++++	nd
France	NCHA000391	La Venotiere	*Myodes glareolus*	neg	+++	nd
France	NCHA000103	Mignovillard	*Myodes glareolus*	++++	neg	Western
France	NCHA000105	Mignovillard	*Myodes glareolus*	++++	++	nd
France	NCHA000124	Mignovillard	*Myodes glareolus*	++++	neg	nd
France	NCHA000127	Mignovillard	*Myodes glareolus*	++++	neg	nd
France	NCHA000106	Mignovillard	*Myodes glareolus*	++++	neg	nd
France	NCHA000130	Mignovillard	*Myodes glareolus*	++++	++++	nd
France	NCHA000113	Mignovillard	*Myodes glareolus*	+++	neg	nd
France	NCHA000132	Mignovillard	*Myodes glareolus*	+++	neg	nd
France	NCHA000090	Mignovillard	*Myodes glareolus*	++	++++	nd
France	NCHA000094	Mignovillard	*Myodes glareolus*	neg	++	nd
France	NCHA000129	Mignovillard	*Myodes glareolus*	neg	++	nd
France	NCHA000010	Mont-sous-Vaudrey	*Myodes glareolus*	++++	neg	Western
France	NCHA000043	Mont-sous-Vaudrey	*Myodes glareolus*	++++	++	nd
France	NCHA000019	Mont-sous-Vaudrey	*Myodes glareolus*	++++	neg	nd
France	NCHA000011	Mont-sous-Vaudrey	*Myodes glareolus*	++++	+++	Western
France	NCHA000004	Mont-sous-Vaudrey	*Myodes glareolus*	++++	neg	nd
France	NCHA000014	Mont-sous-Vaudrey	*Myodes glareolus*	++++	neg	nd
France	NCHA000026	Mont-sous-Vaudrey	*Myodes glareolus*	++++	neg	nd
France	NCHA000025	Mont-sous-Vaudrey	*Myodes glareolus*	++++	neg	Western
France	NCHA000041	Mont-sous-Vaudrey	*Myodes glareolus*	+++	neg	nd
France	NCHA000347	Vouzon	*Myodes glareolus*	++++	neg	Western
France	NCHA000346	Vouzon	*Myodes glareolus*	++++	++++	nd
France	NCHA000373	Vouzon	*Myodes glareolus*	++++	neg	nd
France	NCHA000350	Vouzon	*Myodes glareolus*	++++	++++	nd
France	NCHA000374	Vouzon	*Myodes glareolus*	++++	+++	nd
France	NCHA000380	Vouzon	*Myodes glareolus*	++++	+++	nd
France	NCHA000366	Vouzon	*Myodes glareolus*	+++	++++	nd
France	NCHA000341	Vouzon	*Myodes glareolus*	++	++++	nd
France	NCHA000361	Vouzon	*Myodes glareolus*	neg	++++	nd
France	NCHA000356	Vouzon	*Myodes glareolus*	neg	+++	nd
France	NCHA000359	Vouzon	*Myodes glareolus*	neg	+++	nd
France	NCHA000372	Vouzon	*Myodes glareolus*	neg	++	nd
France	NCHA000370	Vouzon	*Myodes glareolus*	neg	++	nd
France	NCHA000376	Vouzon	*Myodes glareolus*	neg	++	nd
Great Britain	KS15/511	Cumbria	*Myodes glareolus*	neg	++++	nd
Great Britain	KS15/510	Cumbria	*Myodes glareolus*	neg	+++	nd
Great Britain	KS15/531	Pentland Hills	*Myodes glareolus*	neg	++	nd
Great Britain	KS15/533	Pentland Hills	*Myodes glareolus*	neg	++	nd
Great Britain	KS15/526	Pentland Hills	*Myodes glareolus*	neg	++	nd
Italy	74F27B6	Trento	*Myodes glareolus*	++++	neg	Western
Italy	74F0610	Trento	*Myodes glareolus*	++++	neg	nd
Italy	74FD0F2	Trento	*Myodes glareolus*	++++	neg	Western
Italy	728E6E8	Trento	*Myodes glareolus*	neg	++	nd
Italy	74F3312	Trento	*Myodes glareolus*	neg	(+)	nd

The Netherlands	12-1593	Nutter	*Myodes glareolus*	++++	neg	nd
The Netherlands	13-1760	Nutter	*Myodes glareolus*	++++	neg	nd
The Netherlands	12-1640	Nutter	*Myodes glareolus*	++++	++	nd
The Netherlands	12-1580	Nutter	*Myodes glareolus*	++++	++	nd
The Netherlands	12-1605	Nutter	*Myodes glareolus*	++++	neg	nd
The Netherlands	13-1758	Nutter	*Myodes glareolus*	++++	neg	nd
The Netherlands	12-1648	Nutter	*Myodes glareolus*	++++	neg	nd
The Netherlands	13-1759	Nutter	*Myodes glareolus*	++++	neg	nd
The Netherlands	13-1740	Nutter	*Myodes glareolus*	++++	neg	Western
The Netherlands	12-1578	Nutter	*Myodes glareolus*	++++	neg	nd
The Netherlands	13-1761	Nutter	*Myodes glareolus*	+	neg	nd
The Netherlands	12-1632	Nutter	*Myodes glareolus*	neg	++	nd
The Netherlands	12-1629	Nutter	*Myodes glareolus*	neg	+	nd
Austria	Mu/08/1298	Laa a.d. Thaya	*Myodes glareolus*	++++	++	Carpathian
Austria	Mu/08/1241	Laa a.d. Thaya	*Myodes glareolus*	++++	neg	nd
Austria	Mu/08/1264	Laa a.d. Thaya	*Myodes glareolus*	+++	neg	nd
Austria	Mu/08/1265	Laa a.d. Thaya	*Myodes glareolus*	neg	++++	nd
Austria	Mu/08/1221	Laa a.d. Thaya	*Myodes glareolus*	neg	++++	nd
Austria	Mu/08/1266	Laa a.d. Thaya	*Myodes glareolus*	neg	++++	nd
Austria	Mu/08/1290	Laa a.d. Thaya	*Myodes glareolus*	neg	++++	nd
Austria	Mu/08/1220	Laa a.d. Thaya	*Myodes glareolus*	neg	++++	nd
Austria	Mu/08/1288	Laa a.d. Thaya	*Myodes glareolus*	neg	++++	nd
Austria	Mu/08/1302	Laa a.d. Thaya	*Myodes glareolus*	neg	++++	nd
Austria	Mu/08/1238	Laa a.d. Thaya	*Myodes glareolus*	neg	+++	nd
Austria	Mu/08/1285	Laa a.d. Thaya	*Myodes glareolus*	neg	+++	nd
Poland	KS13/863	Dobskie Lake	*Myodes glareolus*	neg	+	Eastern
Sweden	76	Grimsö	*Myodes glareolus*	neg	++	nd
Sweden	61	Grimsö	*Myodes glareolus*	neg	++	nd
Sweden	63	Grimsö	*Myodes glareolus*	neg	++	nd
Sweden	71	Grimsö	*Myodes glareolus*	neg	+	nd
Sweden	323	Haparanda	*Myodes glareolus*	neg	++	nd
Sweden	322	Haparanda	*Myodes glareolus*	neg	++	nd
Sweden	166	Harads	*Myodes glareolus*	neg	++++	nd
Sweden	168	Harads	*Myodes glareolus*	neg	++++	nd
Sweden	163	Harads	*Myodes glareolus*	neg	+++	nd
Sweden	403	Öster Malma	*Myodes glareolus*	neg	++++	nd
Sweden	106	Umea	*Myodes glareolus*	neg	++	Ural
Sweden	109	Umea	*Myodes glareolus*	neg	++	Ural
Sweden	425	Växjö	*Myodes glareolus*	neg	++++	nd
Sweden	412	Växjö	*Myodes glareolus*	neg	++++	nd
Sweden	419	Växjö	*Myodes glareolus*	neg	++++	nd
Sweden	438	Växjö	*Myodes glareolus*	neg	++	nd
Sweden	52	Vindeln	*Myodes glareolus*	neg	++	nd
Slovakia	178	Bratislava	*Myodes glareolus*	++++	neg	nd
Slovakia	140	Bratislava	*Myodes glareolus*	++++	neg	nd
Slovakia	4	Bratislava	*Myodes glareolus*	++++	++++	nd
Slovakia	144	Bratislava	*Myodes glareolus*	++++	neg	nd
Slovakia	146	Bratislava	*Myodes glareolus*	+++	neg	nd
Slovakia	78	Bratislava	*Myodes glareolus*	+++	neg	nd
Slovakia	185	Bratislava	*Myodes glareolus*	+++	neg	nd
Slovakia	173	Bratislava	*Myodes glareolus*	+++	neg	nd
Slovakia	150	Bratislava	*Myodes glareolus*	neg	++++	nd
Slovakia	77	Bratislava	*Myodes glareolus*	neg	++++	nd
Slovakia	62	Bratislava	*Myodes glareolus*	neg	++++	Western
Slovakia	57	Bratislava	*Myodes glareolus*	neg	++++	nd
Slovakia	17	Bratislava	*Myodes glareolus*	neg	++++	nd
Slovakia	101	Bratislava	*Myodes glareolus*	neg	+++	nd
Slovakia	154	Bratislava	*Myodes glareolus*	neg	+++	nd
Slovakia	152	Bratislava	*Myodes glareolus*	neg	+	nd
Slovakia	114	Fugelka	*Myodes glareolus*	++++	neg	nd
Slovakia	137	Fugelka	*Myodes glareolus*	++++	++++	Carpathian
Slovakia	133	Fugelka	*Myodes glareolus*	++++	neg	nd
Slovakia	125	Fugelka	*Myodes glareolus*	+++	neg	nd
Slovakia	216	Fugelka	*Myodes glareolus*	+++	neg	nd
Slovakia	217	Fugelka	*Myodes glareolus*	+++	++++	nd
Slovakia	220	Fugelka	*Myodes glareolus*	+++	neg	nd
Slovakia	117	Fugelka	*Myodes glareolus*	++	neg	nd
Slovakia	13	Fugelka	*Myodes glareolus*	++	+++	nd
Slovakia	124	Fugelka	*Myodes glareolus*	+	neg	nd
Slovakia	2	Fugelka	*Myodes glareolus*	neg	++++	nd
Slovakia	1	Fugelka	*Myodes glareolus*	neg	++++	nd
Slovakia	219	Fugelka	*Myodes glareolus*	neg	++++	nd
Slovakia	16	Fugelka	*Myodes glareolus*	neg	+++	nd

Slovakia	22869	Rozhanovce	*Myodes glareolus*	++++	++++	nd
Slovakia	22867	Rozhanovce	*Myodes glareolus*	++++	neg	nd
Slovakia	22453	Rozhanovce	*Myodes glareolus*	++++	neg	nd
Slovakia	22596	Rozhanovce	*Myodes glareolus*	+++	++++	nd
Slovakia	22505	Rozhanovce	*Myodes glareolus*	+++	++++	nd
Slovakia	22455	Rozhanovce	*Myodes glareolus*	++	+++	nd
Slovakia	22568	Rozhanovce	*Myodes glareolus*	neg	++++	nd
Slovakia	22600	Rozhanovce	*Myodes glareolus*	neg	++++	Eastern
Slovakia	22598	Rozhanovce	*Myodes glareolus*	neg	+++	nd
Slovakia	22866	Rozhanovce	*Myodes glareolus*	neg	+++	nd
Slovakia	22864	Rozhanovce	*Myodes glareolus*	neg	+++	nd
Slovakia	22533	Rozhanovce	*Myodes glareolus*	neg	++	nd

++++, ct-values 20-25; +++, ct-values 25-30; ++, ct-values 30-35; +, ct-values 35-40; (+), borderline positive result (ct-values 40.46 and 41.13); neg, no viral RNA was detected; nd, not determined

Table S3: Nucleotide (nt) and amino acid (aa) sequence identity of BvHV sequences to sequences of the same trapping site and to reference sequences of species *Hepacivirus J* and *Hepacivirus F*.

	Trapping site	% nt / aa identity within trapping site and same clade	% nt / aa identity within trapping site between clades	% nt / aa identity with reference KC411777 *Hepacivirus J*	% nt / aa identity with reference KC411784 *Hepacivirus F*
Hepacivirus J	Laa an der Thaya	- / -	- / -	83.01 / 98.69	55.12 / 54.25
	Cormaranche en Bugey	94.55 / 100	- / -	84.31-86.06 / 98.69	57.30-57.73 / 54.25
	La Venotiere	84.53-89.32 / 98.69-99.35	- / -	81.92-83.88 / 96.39-96.73	54.25-53.81 / 52.94
	Mignovillard	83.22-100 / 96.73-100	- / -	83.88-86.71 / 96.73-97.39	53.81-55.77 / 52.94-53.59
	Mont sous Vaudrey	87.36-94.12 / 95.42-100	- / -	82.35-86.49 / 95.42-98.69	54.25-56.43 / 52.94-54.25
	Vouzon	- / -	- / -	81.48 / 95.42	53.59 / 51.63
	Jasnitz	- / -	- / -	84.53 / 98.04	54.03 / 54.25
	Osnabrück	85.84 / 99.35	- / -	82.14-84.31 / 97.39-98.04	54.90-55.34 / 54.25-54.90
	Schrevendorf	- / -	- / -	84.10 / 98.04	55.34 / 54.25
	Trento	90.85-99.35 / 99.35-100	- / -	83.66-84.75 / 98.04	55.12-56.21 / 54.25-54.90
	Nutter	94.77-100 / 100	- / -	84.75-85.19 / 98.69	54.90-55.34 / 54.25
	Bratislava	90.20-91.94 / 98.04-100	56.21-57.52 /55.56	80.39-82.57 / 97.39-98.69	54.03-57.52 / 54.25
	Rozhanovce	- / -	53.59-53.81 /55.56	83.88 / 98.69	54.47 / 54.25
	Fugelka	88.89-99.35 / 98.69-10	- / -	79.74-81.92 / 98.04-98.69	54.90-56.21 / 54.25-54.90
Hepacivirus F	Hangenleithen	- / -	- / -	53.81 / 55.56	79.08 / 94.77
	Mühlberg	- / -	- / -	55.56 / 55.56	79.30 / 95.42
	Raimundsreuth	- / -	- / -	53.81 / 55.56	79.74 / 95.42
	Rothemühl	99.35 / 98.69	- / -	53.16-53.38 / 78.65-79.08	78.65-79.08 / 92.16-93.46
	Tussenhausen	- / -	- / -	53.16 / 55.56	77.12 / 93.46
	Bratislava	- / -	56.2-57.5 / 55.56	55.77 / 55.56	81.05 / 94.77
	Rozhanovce	95.86 / 99.35	53.59-53.81 / 55.56	52.29-52.51 / 55.56	79.96-80.17 / 95.42-96.08

- / -, only one sequence of this species at the trapping site.

1. Drexler, J.F.; Corman, V.M.; Müller, M.A.; Lukashev, A.; Gmyl, A.; Coutard, B.; Adam, A.; Ritz, D.; Leijten, L.M.; Van Riel, D. et al. Evidence for novel hepaciviruses in rodents. PLoS Pathog. 2013;9(6):e1003438 DOI: 10.1371/journal.ppat.1003438.
2. Röhrs, S.; Begeman, L.; Straub, B.K.; Boadella, M.; Hanke, D.; Drewes, S.; Hoffmann, B.; Keller, M.; Drexler, J.F.; Drosten, C. et al. The bank vole (*Clethrionomys glareolus*) - small animal model for hepacivirus infection. submitted.
3. Hoffmann, B.; Depner, K.; Schirrmeier, H.; Beer, M. A universal heterologous internal control system for duplex real-time RT-PCR assays used in a detection system for pestiviruses. J Virol Methods. 2006;136(1-2):200-9 DOI: 10.1016/j.jviromet.2006.05.020.
